# Network and biosignature analysis for the integration of transcriptomic and metabolomic data to characterize leaf senescence process in sunflower

**DOI:** 10.1186/s12859-016-1045-2

**Published:** 2016-06-06

**Authors:** Sebastián Moschen, Janet Higgins, Julio A. Di Rienzo, Ruth A. Heinz, Norma Paniego, Paula Fernandez

**Affiliations:** Instituto de Biotecnología, Centro de Investigaciones en Ciencias Agronómicas y Veterinarias, Instituto Nacional de Tecnología Agropecuaria, Hurlingham, Buenos Aires, Argentina; Consejo Nacional de Investigaciones Científicas y Técnicas, Ciudad Autónoma de Buenos Aires, Argentina; The Genome Analysis Centre, Norwich Research Park, Norwich, NR4 7UH UK; Facultad de Ciencias Agropecuarias, Universidad Nacional de Córdoba, Córdoba, Argentina; Escuela de Ciencia y Tecnología, Universidad Nacional de San Martín, San Martín, Buenos Aires, Argentina

**Keywords:** WGCNA, BioSignature Discoverer, Leaf senescence, Transcriptomic, Metabolomic, Data integration, Sunflower

## Abstract

**Background:**

In recent years, high throughput technologies have led to an increase of datasets from omics disciplines allowing the understanding of the complex regulatory networks associated with biological processes. Leaf senescence is a complex mechanism controlled by multiple genetic and environmental variables, which has a strong impact on crop yield. Transcription factors (TFs) are key proteins in the regulation of gene expression, regulating different signaling pathways; their function is crucial for triggering and/or regulating different aspects of the leaf senescence process. The study of TF interactions and their integration with metabolic profiles under different developmental conditions, especially for a non-model organism such as sunflower, will open new insights into the details of gene regulation of leaf senescence.

**Results:**

Weighted Gene Correlation Network Analysis (WGCNA) and BioSignature Discoverer (BioSD, Gnosis Data Analysis, Heraklion, Greece) were used to integrate transcriptomic and metabolomic data. WGCNA allowed the detection of 10 metabolites and 13 TFs whereas BioSD allowed the detection of 1 metabolite and 6 TFs as potential biomarkers. The comparative analysis demonstrated that three transcription factors were detected through both methodologies, highlighting them as potentially robust biomarkers associated with leaf senescence in sunflower.

**Conclusions:**

The complementary use of network and BioSignature Discoverer analysis of transcriptomic and metabolomic data provided a useful tool for identifying candidate genes and metabolites which may have a role during the triggering and development of the leaf senescence process. The WGCNA tool allowed us to design and test a hypothetical network in order to infer relationships across selected transcription factor and metabolite candidate biomarkers involved in leaf senescence, whereas BioSignature Discoverer selected transcripts and metabolites which discriminate between different ages of sunflower plants. The methodology presented here would help to elucidate and predict novel networks and potential biomarkers of leaf senescence in sunflower.

**Electronic supplementary material:**

The online version of this article (doi:10.1186/s12859-016-1045-2) contains supplementary material, which is available to authorized users.

## Background

Functional genomics is a field of molecular biology that studies the functions and interactions between genes and proteins from large datasets generated by genome projects. Over recent years, the development of new high throughput technologies among different *omics* approaches has led to the availability of a large volume of transcriptomic, metabolomic, proteomic, physiological and phenotypic data. In this sense, the multidisciplinary synergy between molecular biology, statistics and informatics emerge as necessary to support the use and interpretation of the results in the area of functional genomics in order to provide specific tools for the simultaneous analysis of many genes.

Senescence is the final stage of leaf development and precedes cell death. Once the senescence program is triggered, important highly regulated gene expression changes occur, leading to important changes in the metabolism [1–7].

In sunflower, which is the fourth most important oil crop worldwide, a delay in leaf senescence has a strong impact on yield; this effect has also been shown in other crops. Maintaining the photosynthetic leaf area especially during the reproductive stage [8–12] has been shown to impact on the gap between potential and real yield observed, this is due to the incapacity of plants to maintain their green leaf area for longer periods [13, 14].

Transcription factors (TFs) are key proteins involved in the regulation of gene expression and signal transduction networks, regulating different biological processes. Several transcription factor families have been associated with leaf senescence in a range of species [15–29] and their function is crucial for triggering and/or regulating the different aspect of this process.

Various software tools have been developed with the aim of analyzing datasets to predict hub genes which could regulate a specific pathway or be involved in a particular biological process. Weighted Gene Correlation Network Analysis (WGCNA) is an R package method designed to find clusters (modules) of highly correlated genes or metabolites. WGCNA calculates a Pearson’s correlation matrix for all the genes and then transforms the correlation matrix into an adjacency matrix by raising all values to a soft threshold power; this has the effect of emphasizing the strong correlations and penalizing the weaker correlations. The *module eigengene (ME)* is the first principal component of a given module and can be considered as representative of the module’s gene expression profile. Modules often represent specific biological processes; highly connected hub genes within the module are often regulatory genes and represent candidate biomarker. WGCNA can be used to construct networks in which each node represents a gene or metabolite and the connecting lines (edges) represent correlations between the nodes. WGCNA has been widely used as a method to cluster gene expression and metabolite data and identify hub biomarker genes in several non-model plant species such as tomato [30], Brassica [31], Petunia [32], rice [33] and Ficus [34].

BioSignature Discoverer (BioSD) is a software application devised for identifying molecular signatures in different biological datasets, such as Next Generation Sequencing, microarray data and metabolic profiles, in a statistically robust, computationally efficient, and user-friendly way. A signature is defined as the minimal set of molecular quantities that collectively yield maximal predictive performance. Thus, a signature does not contain irrelevant or redundant quantities, given the selected ones. In addition, there may be numerous equivalent signatures that lead to equally accurate predictions, employing different molecular quantities. The core of this application uses a feature selection algorithm that belongs to the class of Bayesian Network, constraint-based learning [35] and that is able to identify multiple, statistically equivalent signatures.

In this work we used these two complementary methods, WGCNA and BioSD, with the aim of identifying potential transcription factors and metabolites, as putative biomarkers associated with leaf senescence in sunflower.

## Methods

### Plant material and experimental conditions

The sunflower leaf senescence assays were conducted under field conditions. The experiment was carried out at the INTA Balcarce Experimental Station (37°45’ S, 58°18’ W) as previously described [36, 37]. The experiment was sowed during the 2010/11 growing season. The sunflower hybrid VDH 487 (Advanta Seeds, Argentina) was sown at a 7.2 plants/m2 with three biological replicates (plots), each one consisted of three randomly selected plants from each plot.

Sunflower assay was conducted under control conditions, without limitations (water and nutrients). Transcriptomic and metabolomics profiling was performed using the leaf 10 (numbered from the bottom to the top of the plant) at three different development stages labeled as T-0 (young leaf, 48 days after emergence of the plant with maximum chlorophyll content), T-1 (pre-anthesis leaf, 62 days after emergence of the plant with 80 % of chlorophyll content) and T-2 (post-anthesis leaf, with senescence symptoms, 69 days after emergence of the plant with 50 % of chlorophyll content) [36, 37]. The samples were immediately frozen in liquid nitrogen upon collection and saved at −80 °C until processing. High quality total RNA was isolated from 100 mg of frozen tissue using TriPure, according to the manufacturer’s instructions (Roche, Buenos Aires, Argentina). The genomic DNA was eliminated by treatment with DNase I for 20 min at room temperature (Invitrogen, Buenos Aires, Argentina). The RNA concentration was measured using a Nanodrop ND-1000 spectrophotometer (NanoDrop Technologies, Wilmington, Delaware USA). The purity and integrity of total RNA was determined by 260/280 nm ratio and by NanoBioanalyzer RNA-6000 analysis (Agilent Technologies, Palo Alto, California USA).

### Data set

Transcriptomic profiling was performed using a custom sunflower microarray (Agilent 4x44K format) which has been previously described [38]. Background correction was performed using the *rma* algorithm from the *backgroundCorrect* function (offset = 16, other parameters by default). Between arrays normalization was performed using the *quantile* method from the *normalizeBetweenArray* function. Finally, gene expressions were transformed to log_2_ scale and information from technical replicates was incorporated by calculating the median parameter. Statistically significant probesets where identified using the *lmFit* and *contrasts.fit* functions from the R software *Limma* Bioconductor Package [39, 40] A total of 4,909 probes were shown to be statistically significant during leaf senescence as the plants develop (T-1 *vs.* T-0 and/or T-2 *vs*.T-0). The statistical parameters were p-value lower than 0.05 and fold-change higher or lower than 2 [37]. Sunflower transcription factors (TFs) were identified by comparing approximately 23,000 TFs sequences from *Arabidopsis lyrata, Arabidopsis thaliana, Oryza sativa, Populus trichocarpa, Vitis vinifera* and *Zea mays* available from the Plant Transcription Factor Database (http://plntfdb.bio.uni-potsdam.de/v3.0/) [41] with SUR v1.0 database [38] using Blast software. A total of 82 TFs were differentially expressed during leaf senescence.

Metabolic profiling was performed using the GC-TOF-MS system (LECO Corporation, St. Joseph, Michigan, USA). Metabolite extraction was performed by promoting the extraction of lipophilic and polar compounds according to recently published protocols [42] adapted for sunflower tissue samples [43]. The chromatograms and spectra were evaluated using ChromaTOF (LECO Corporation, St. Joseph, Míchigan, USA) and TagFinder [44]. Ion spectra were compared to the Golm Metabolome Database (http://gmd.mpimp-golm.mpg.de/). Metabolite levels were normalized to fresh weight using ribitol as the internal control. Changes in metabolite levels along leaf development were calculated as fold-change relative to the first sampled time (T-0).

### Weighted Gene Co-expression network analysis (WGCNA)

WGCNA was performed using the WGCNA R package (v1.42) as described by Langfelder and Horvath [45]. The expression values for 9,592 non-redundant genes and levels of 62 metabolites for the 9 samples were used to construct two separate networks. Samples were clustered using the function *hclust* to check that there were no outliers. The *pickSoftThreshold* function was used to select the soft threshold power used to construct a network based on the criterion of approximate scale-free topology. The power value selected was the lowest power for which the scale-free topology fit index curve flattened out upon reaching a high value. The next step was to transform the adjacency matrix into a topological overlap matrix (TOM), which summarizes the degree of shared connections between any two genes. The TOM matrix was then converted into a dissimilarity matrix. Genes were then clustered using the average linkage hierarchical clustering and the modules were identified in the resulting dendrogram using the dynamic hybrid tree cut method (Additional file 1). Found modules were trimmed of genes whose correlation with the module eigengene (KME) was less than *minKMEtoStay.* If p-values of the higher correlations were smaller than those of the native module by the factor *reassignThreshold,* the gene was re-assigned to the closer module. Modules whose eigengenes were highly correlated were merged. This was achieved by clustering module eigengenes using the dissimilarity given by one minus their correlation, cutting the dendrogram at the height *mergeCutHeight* and merging all modules on each branch below *mergeCutHeight*. This was implemented using the *blockwiseModules* function in WGCNA which performs the network construction and consensus module detection. The following settings were used for the gene network; power = 6, minModuleSize = 50, mergeCutHeight = 0.2, maxBlockSize = 10000, deepSplit = 2, reassignThreshold = 1e-6 and minKMEtoStay = 0.5, networkType = “unsigned”, TOMType = “signed”. The genes were divided into 17 modules; the module sizes ranged from 99 to 2,967 genes, 501 genes were not assigned to any of the modules. The following settings were used for the metabolite network; power = 10, minModuleSize = 5, mergeCutHeight = 0.2, maxBlockSize = 1000, deepSplit = 2, reassignThreshold = 1e-6 and minKMEtoStay = 0.5, networkType = “unsigned”, TOMType = “signed”. The metabolites were divided into 3 modules; the module sizes ranged from 10 to 35 metabolites, 5 metabolites were not assigned to any of the modules.

Module Membership (MM) is a quantitative measure of module membership; this is the correlation of the ME and the gene expression profile for each gene in the module. The gene with the highest connectivity in each module was returned using the function *chooseTopHubInEachModule.*

The networks were exported from WGCNA using the function *exportNetworkToCytoscape.*

The gene network showed approximate scale free topology, whereas the metabolic network did not satisfy scale free topology: 9592 genes scaleFreeRsquared 0.86 slope −0.62 and 62 metabolites scaleFreeRsquared 0.33 slope −0.54 (Additional file 2).

A total of 24 metabolites with a degree higher than 35 were then selected as metabolite hubs and correlated with the list of 82 TFs statistically significant during senescence (Additional file 3) using Pearson correlation. Correlations with p-value <0.0001 were selected and visualized using Cytoscape [46].

### BioSignature Discoverer

The statistical analysis pipeline of BioSD, a plugin of QIAGEN’s CLC-Bio workbench (CLC Bio, Aarhus, Denmark), has been used for identifying gene-expression signatures able to discriminate the age of sunflower plants. Particularly, the plugin has been applied on the expression values of 82 transcription factors that were found differentially expressed during leaf senescence, on the 62 metabolites measured in the study, and on the combination of these two data types. Additionally, the pipeline has been separately applied on the extended list of 9,592 non-redundant genes.

BioSD employs a complex machine-learning approach in order to discover such signatures and for quantifying their actual predictive power. Particularly, this tool employs novel, proprietary feature selection algorithms to identify the signatures, inspired by prior work on Bayesian-Network constraint-based learning [35]. Notably, the pipeline is also able to identify multiple equivalent signatures, whose predictive performances are statistically equivalent. To avoid under fitting, the tool automatically tries multiple algorithms for feature selection and prediction modeling, as well as multiple values for tuning them. The tool also employs sophisticated methods, based on an enhanced version of cross-validation [47], for providing conservative estimations of the performances that can be achieved with the given signatures.

The BioSD interface allows the user to modulate the trade-off between quality of the results and computational requirements through the ‘Tuning Effort’ parameter. This parameter regulates the extent to which the tool will attempt tuning the feature selection and prediction modeling algorithms. In all analyses, the ‘Tuning Effort’ was set to ‘Extensive’, in order to favor result accuracy at the expense of higher computational time.

## Results and discussion

WGCNA is a powerful method used to identify clusters of highly correlated genes which are potentially co-regulated. All the connections for the metabolites with an edge weight >0.1 produced a network of 51 nodes and 765 edges. The connections between the 82 significant differentially expressed transcription factors were selected from the gene network with an edge weight >0.4, producing a network of 75 nodes and 1202 edges.

**Fig. 1:**
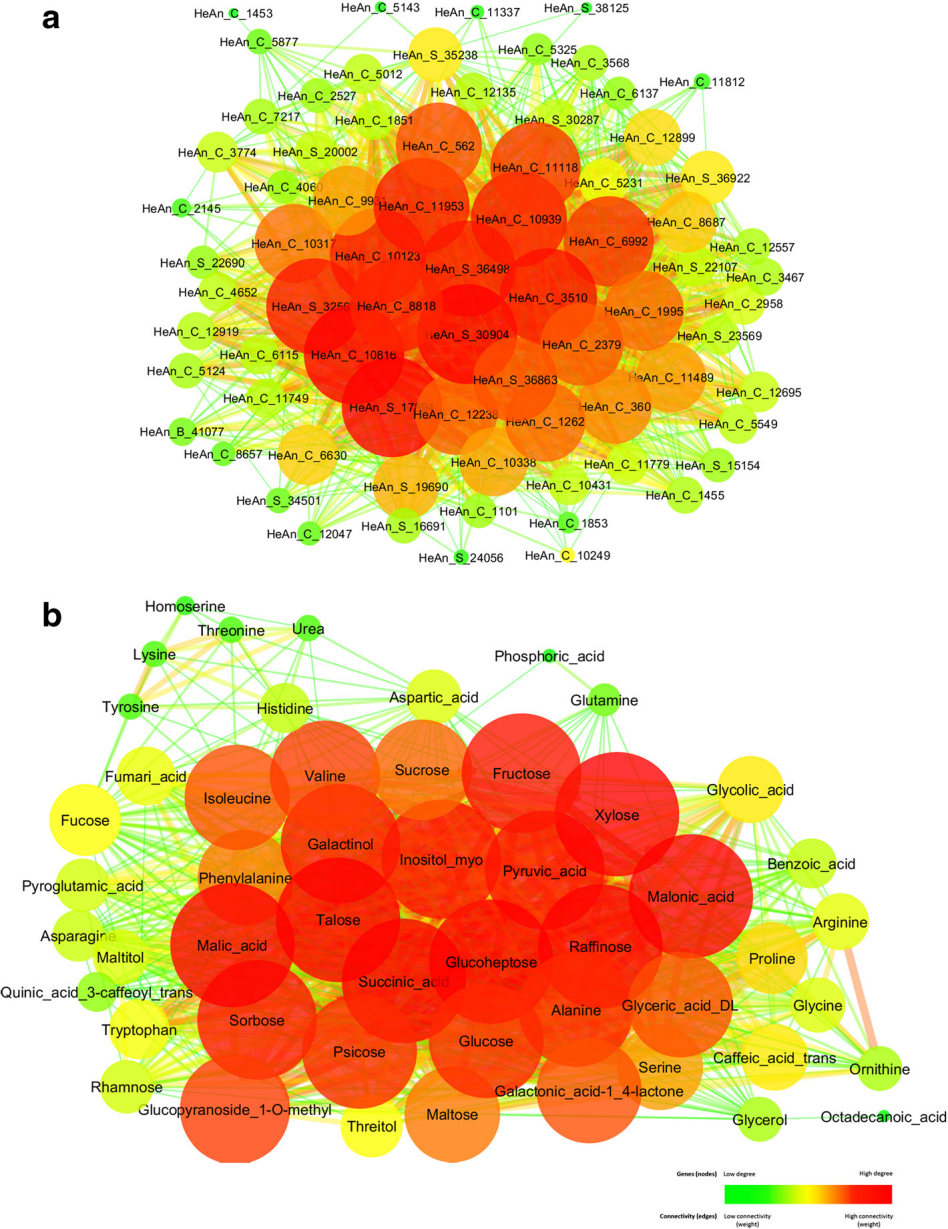
Networks exported from WGCNA visualized in Cytoscape. Network were constructed using 75 transcription factors (**a**) and 51 metabolites (**b**) and were visualized in Cytoscape [46] with an edge weight higher than 0.3. The nodes represent genes and metabolites and the edges represent connections between them. The node size and color is related to the number of connections, large red nodes represent highly connected hub genes and metabolites, small green nodes represent gene with few connections. Strong connections are visualized as wider lines

The networks were visualized in Cytoscape by edge weight and degree (Fig. 1a and b).

A total of 20 transcription factors and 24 metabolites showed the highest number of connections (degree), over 80 % of the maximum number of connections observed (degree >60 for TFs and degree >35 for metabolites), highlighting them as potentially co-regulated hub biomarkers during the senescence process in sunflower.

**Fig. 2:**
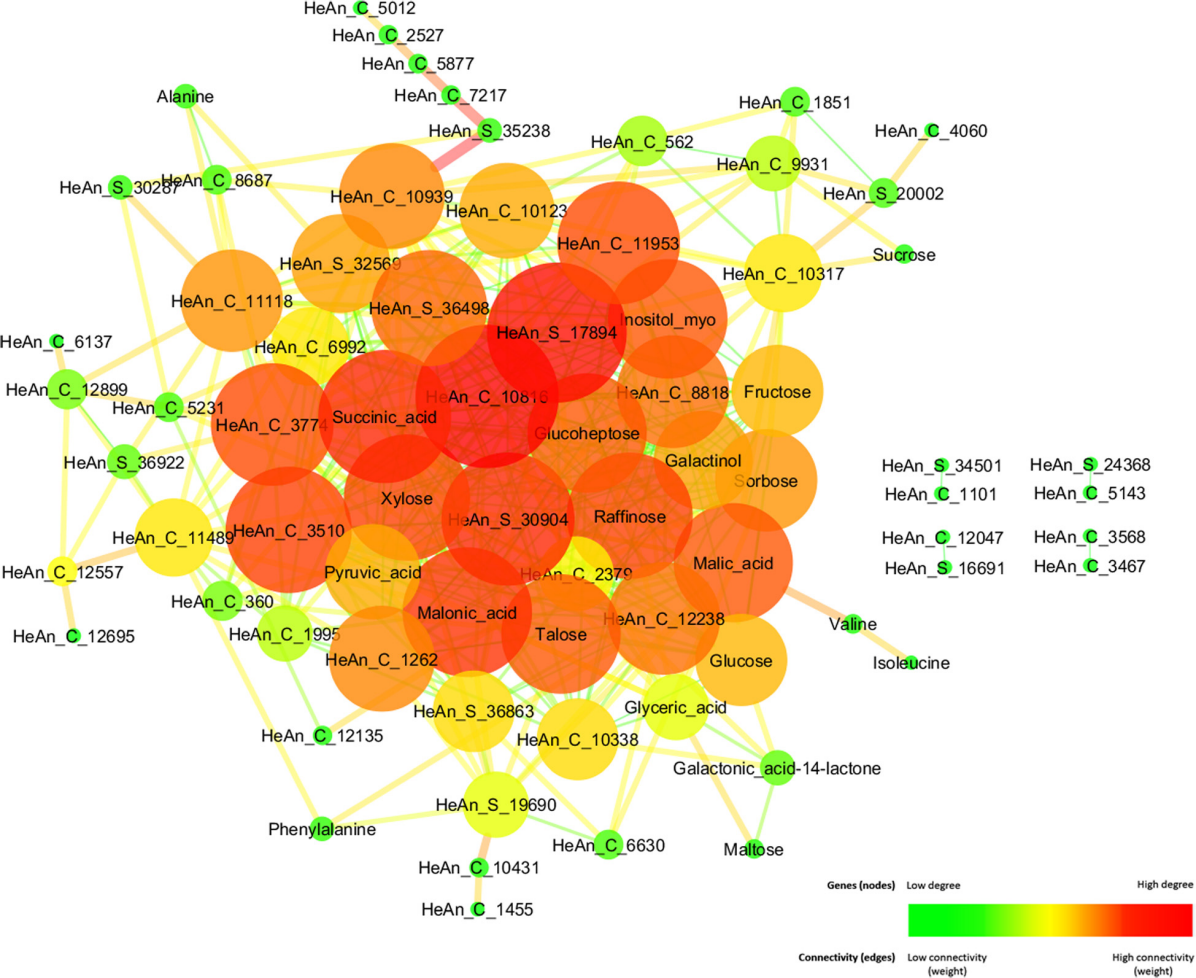
Integrated network of hubs metabolites and transcription factors. Pearson correlation analysis of 24 selected hubs metabolites and the list of 82 TFs statistically significant during senescence. Correlations with p-value < 0.0001 were selected and visualized in Cytoscape [46] by degree (node size and color) and edge weight (edge size and color)

Pearson correlation analysis between the 24 hub metabolites and the list of 82 TFs significant during senescence was performed in order to integrate both analyses. The resulting network contained 74 nodes and 455 edges (Fig. 2).

The integration results showed that 7 metabolite hubs correspond to sugars and TCA cycle metabolites(from a total of 10) and the NAC TFs family, widely reported as senescence associated, showed 3 members (from a total of 13) as hub genes.

On the other hand, BioSD allows identification of the most informative genes and metabolites for discriminating between the various stages of the leaf senescence process. Table 1 lists all the transcription factors and metabolites whose values allow the predicting of senescence in sunflower plants. All these quantities have equivalent discriminative power and 100 % stability, which mean that they are consistently selected even when other datasets are included.

**Table 1:** List of biosignatures detected in each dataset

Metabolites	Transcription factors	Integrated list
Alanine	HeAn_C_10939	Alanine
	HeAn_C_11118	HeAn_S_15792
	HeAn_C_11953	HeAn_C_10939
	HeAn_C_12899	HeAn_S_38523
	HeAn_C_8526	HeAn_C_9238
	HeAn_C_9238	HeAn_S_32569
	HeAn_S_15792	HeAn_C_11118
	HeAn_S_32569	
	HeAn_S_36498	
	HeAn_S_38523	

Each signature is composed of a single biomarker, and all signatures are expected to have the same predictive performance (see Table 2)

Table 2 reports the ‘in-sample’ and ‘out-of-sample’ performances of the selected transcripts and metabolites. The in-sample values quantify the fitness of the predictive models, and poor results would indicate presence of outliers, unmeasured yet needed predictive factors, or poorly modeled trends in the data. The out-of-sample values estimate the performances that are expected if the selected expression and metabolic values are used for predicting the age of new, independent samples of sunflower plants (provided the new samples come from the same population of the training data). Assuming *y* and *ŷ* are the observed and predicted age of *n* plants. The *R*^2^ metric measures the proportion of *y* variance explained by the predictive model, *R*^2^ values close to 1 are indicative of good fit, while the Mean Absolute Error (MAE) and Mean Squared Error (MSE) statistics quantify predictions’ deviations from the actual age as $MAE=\frac{1}{n}\cdot {\displaystyle \sum } abs\left(y-\widehat{y}\right)$ and $MSE=\frac{1}{n}\cdot {\displaystyle \sum }{\left(y-\widehat{y}\right)}^2$.

**Table 2:** Predicted performances of the selected biosignatures

	Metric	In Sample	Out Sample	95 % Confidence Interval
Metabolites	R^2^	0.997	0.840	[ 0.335, 0.972 ]
	Mean Absolute Error	0.228	2.503	[ 0.783, 4.060 ]
	Mean Squared Error	0.2104	12.180	[ 0.964, 24.916 ]
Transcription factors	R^2^	0.983	0.825	[ 0.565, 0.939 ]
	Mean Absolute Error	0.897	2.658	[ 1.009, 4.950 ]
	Mean Squared Error	1.239	13.315	[ 3.743, 29.804 ]
Integrated list	R^2^	0.9965	0.909	[ 0.721, 0.986 ]
	Mean Absolute Error	0.465	1.779	[ 0.487, 3.919 ]
	Mean Squared Error	0.2639	6.927	[ 0.469, 18.832 ]

Performances are reported in terms of the determination coefficient R^2^, Mean Absolute Error (MAE) and Mean Squared Error (MSE, see text for more details on these metrics). The in-sample performances quantify the fitness of the predictive models on the training data, while the out-of-sample values estimate the expected performance on new data. Confidence intervals are calculated using a bootstrap approach

In the present application both in- and out-of-sample performances show quite good results, indicating (a) that the predictive models adequately fit the available samples and (b) that the selected transcript factors and metabolites have a high predictive power. MAE, a robust estimator of the standard deviation, is around 2.5 days for the “out of sample” for both, metabolite and transcript data, meaning that it should be possible to predict the age of a new plant on the basis of transcriptional or metabolic information with an average error of approximately three days.

Residual vs. predicted value plots did not showed any apparent pattern or trend, indicating that the models have fitted the data adequately.

The analysis of the complete list of gene (9,592 non-redundant sunflower genes) integrated with metabolic information identified twelve distinct signatures each composed of two elements. Particularly, each signature contains the gene HeAn_C_267, coupled either with the Alanine metabolite or with one of the following eleven genes: HeAn_C_1048, HeAn_C_11045, HeAn_C_11058, HeAn_C_11653, HeAn_C_243, HeAn_C_3359, HeAn_C_8838, HeAn_S_19086, HeAn_S_20155, HeAn_S_30642, HeAn_S_35632. These signatures have slightly better performances than the single biomarkers reported in Table 1 (see Additional file 4).

These results indicate that the Alanine metabolite is interchangeable, in terms of predictive power with each of the other eleven genes. Thus, practitioners interested in deploying this signature in practice (e.g., researchers, agronomists) can freely decide whether to build a predictive model using exclusively transcriptional information or a mixture of transcriptional and metabolic data. This decision can depend on multiple factors, particularly considerations about the cost and technical practicability of measuring the quantities included in the signature.

In recent years, many studies have focused on the detection of different genes up or down-regulated along the progression of the leaf senescence process, identifying TFs that could be triggering the process [1, 3, 29, 48–51]. Hence, studying interactions between these TFs under different conditions, for a non-model organism, will open new insights into the details of gene regulation by identifying new interactions and comparing them to those already observed in model organisms [52].

One of the most important aims in new generation biotech crops is to increase the yield and to improve tolerance to different stresses. Although sequencing-based approaches are becoming the method of choice for gene expression profiling compared to hybridization-based ones, for non-model species for which a reference sequence is not available, the hybridization approach can still be the more reliable method [53].

This study reports on the use of two complementary software applications with the aim of producing a powerful network and predicting potential biosignatures enabling the identification of important TFs and metabolites involved during the leaf senescence process in sunflower.

Using WGCNA analysis, genes and metabolites are clustered in one or more modules. Genes in each module are highly correlated, and consequently they are likely to be co-regulated during the senescence process. BioSD identifies which genes are the most informative in order to discriminate between different stages of leaf development. The selected genes, in this case, are not necessarily correlated among themselves.

In other words, WGCNA attempts to identify the elements involved in the senescence process, and to gain insight of their interplay. On the other hand, BioSD tries to retain the minimum number of these elements that are needed for optimal prediction of the plant senescence process.

WGCNA allowed the detection of metabolite hubs during leaf senescence, most of them correspond to sugars and TCA cycle metabolites. Sugars are central elements in the sink-source relationships [54, 55] and have been reported as growth [56] and photosynthesis regulators [57]. Sunflower is a crop plant with a strong demand for nutrients, especially sugar as a substrate in the oil synthesis during grain filling; this could potentially act as a trigger signal for the senescence process.

BioSD allowed the detection of one metabolite and ten TFs as potential biomarkers. The selected biosignatures are able to estimate the senescence of a plant with an error of approximately 2.5 days. When both TF and metabolite data are simultaneously analyzed, the expected error decreases to 1.8 days, indicating that the integration of different omic data allows better predictions of sunflower senescence by BioSD analysis.

Transcription factors are major players and some of them constitute major hubs in signaling pathways. In both analyses, the selected genes belong to transcription factor families widely reported as senescence associated in model species.

When we analyze the integration of metabolites and TFs (Table 3) we found that three TFs (*HeAn_C_10939, HeAn_C_11118* and *HeAn_S_32569*) are detected as biomarkers independently in the two methods. The expression profiles of the selected biomarkers during leaf senescence are displayed in the Additional file 5. *HeAn_C_10939* presents high sequence similarity to *ATERF3* (AT1G50640) which encodes a member of the ERF (ethylene response factor).

**Table 3:** Candidate biomarkers detected by the two complementary methods, WGCNA and BioSignature Discoverer

WGCNA			Biosignatures		
Sunflower ID	Arabidopsis ID	TF Family	Sunflower ID	Arabidopsis ID	TF Family
HeAn_C_10816	AT1G52890	NAC	HeAn_S_15792	AT5G26210	Alfin-like
HeAn_S_17894	AT5G55580	mTERF	**HeAn_C_10939**	**AT1G50640**	**AP2**
HeAn_C_3510	AT4G27410	NAC	HeAn_S_38523	AT3G16770	AP2
HeAn_S_30904	AT4G17810	C2H2	HeAn_C_9238	AT2G39770	GRAS
HeAn_C_3774	AT4G02590	bHLH	**HeAn_S_32569**	**AT5G49620**	**MYB**
HeAn_C_11953	AT2G45650	MADS	**HeAn_C_11118**	**AT1G69490**	**NAC**
HeAn_S_36498	AT2G30400	OFP	Alanine		
**HeAn_C_10939**	**AT1G50640**	**AP2**			
HeAn_C_1262	AT1G64860	Sigma70-like			
HeAn_C_8818	AT2G28810	C2C2-Dof			
**HeAn_C_11118**	**AT1G69490**	**NAC**			
HeAn_C_12238	AT1G27320	Orphans			
**HeAn_S_32569**	**AT5G49620**	**MYB**			
Succinic_acid					
Malonic_acid					
Raffinose					
Xylose					
Talose					
Inositol_myo					
Glucoheptose					
Malic_acid					
Galactinol					
Sorbose					

Biomarkers from WGCNA were selected based on their degree (higher than 20) and biomarkers from BioSignature Discoverer correspond to the analysis of the integrated list. Biomarkers detected independently in the two methods are in bold

Transgenic Arabidopsis plants with enhanced expression of this TF showed precocious leaf senescence [25]. Additionally, *HeAn_C_11118* has high sequence similarity to *AtNAP/ANAC029* (AT1G69490), a NAC transcription factor which acts downstream of *EIN2* and *EIN3* genes. EIN3 positively regulates leaf senescence by activating *ORE1* and *AtNAP.* Genetic analysis suggest that both genes act in distinct and overlapping signaling pathways regulating leaf senescence in Arabidopsis [17, 58]. *HaNAC01*, a putative sunflower orthologous gene to *ORE1*, has previously been reported in sunflower as a candidate biomarker for leaf senescence [36], which reinforces these genes as potential regulators of the senescence process in sunflower.

*HeAn_S_32569* has high sequence similarity to *MYB78* (AT5G49620). This TF has not yet been directly associated with the senescence process, however, members of this family have been reported as associated to senescence and involved in response to several abiotic stresses [21, 22, 59].

These results suggest that WGCNA and the identification of molecular biosignatures are powerful tools for the detection of potential biomarkers. However, it should be noted that these results are obtained on a relatively small experiment (3 biological replicates for 3 treatments). Further studies would be advisable in order to better estimate the predictive performance of this approach and to achieve best fit for the list of candidate *omics* signatures.

The implementation of data integration analysis associated to different biotic and abiotic stresses is a powerful tool, especially in a non-model crop such as sunflower for which the complete genome sequence is not yet available. These results open new strategies of analysis to explore and detect potential biomarkers associated with leaf senescence that will be useful for future molecular breeding programs.

## Conclusions

The complementary analysis of transcriptomic and metabolomic data with WGCNA and BioSD emerge as a useful strategy to predict not only the age of the plants but also to detect and identify potential biomarkers associated with leaf senescence. In the case of sunflower, a worldwide oil crop, the implementation of WGCNA enabled the construction of a hypothetical network which was used to infer relationships between TFs and metabolites and the identification of hubs as potential candidate biomarkers involved in leaf senescence, whereas BioSD selected transcripts and metabolites to discriminate the age of sunflower plants. This methodological approach is a novel strategy for omics data integration, it can be used to elucidate and predict novel networks as well as identifying potential transcripts as putative biomarker hub genes at different developmental stages.

## Additional files

Additional file 1: Cluster dendrogram visualizing the modules from WGCNA. **a** Genes, **b** Metabolites were clustered using the average linkage hierarchical clustering and modules identified in the resulting dendrogram by the dynamic hybrid tree cut method. (DOCX 530 kb) Additional file 2: Scale-free topologies. **a** Genes, **b** Metabolites. The left panel shows a histogram of network connectivity. The right panel shows a log-log plot of the histogram. The approximate straight line relationship (high R2 value) shows approximate scale free topology. (DOCX 143 kb) Additional file 3: List of 24 hubs metabolites and 82 transcription factors. The list shows the identifiers and the normalized expression data of the different samples. (XLSX 21 kb) Additional file 4: BioSignature Discoverer results on predicting senescence from integrated metabolomics and transcriptomics data in sunflower. (DOCX 38 kb) Additional file 5: Expression profiles of the selected biomarkers during leaf senescence. The list shows the identifiers, the expression profile of candidate biomarkers in log2 scale and the standardization of this expression profile. The figures show the genes and the metabolites in red and blue lines respectively. (XLSX 27 kb)
