# Supplementary figures and images for: Network and biosignature analysis for the integration of transcriptomic and metabolomic data to characterize leaf senescence process in sunflower
Source: BMC Bioinformatics. 2016 Jun 6;17(Suppl 5):174. doi: 10.1186/s12859-016-1045-2 (PMC4905614; doi:10.1186/s12859-016-1045-2)

a


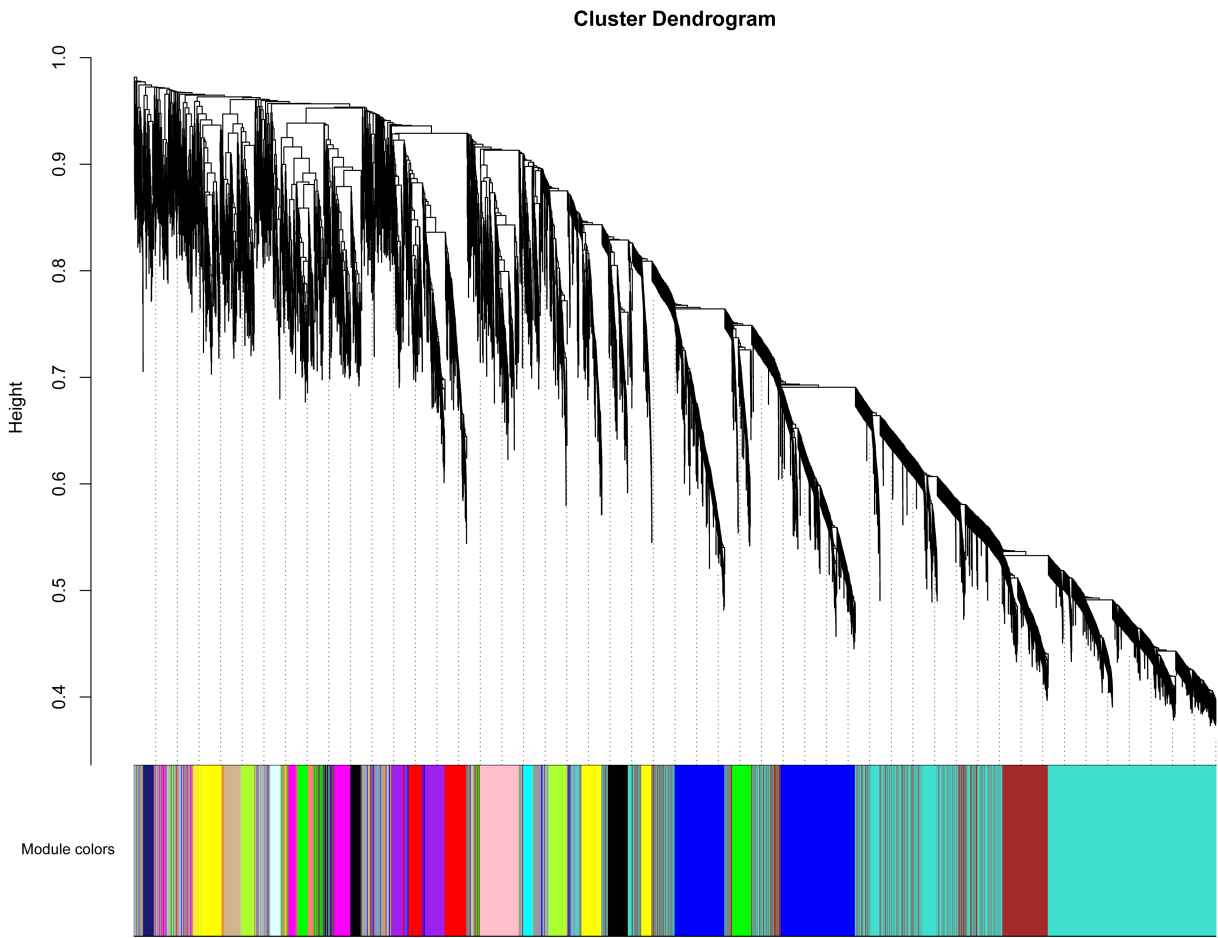


b


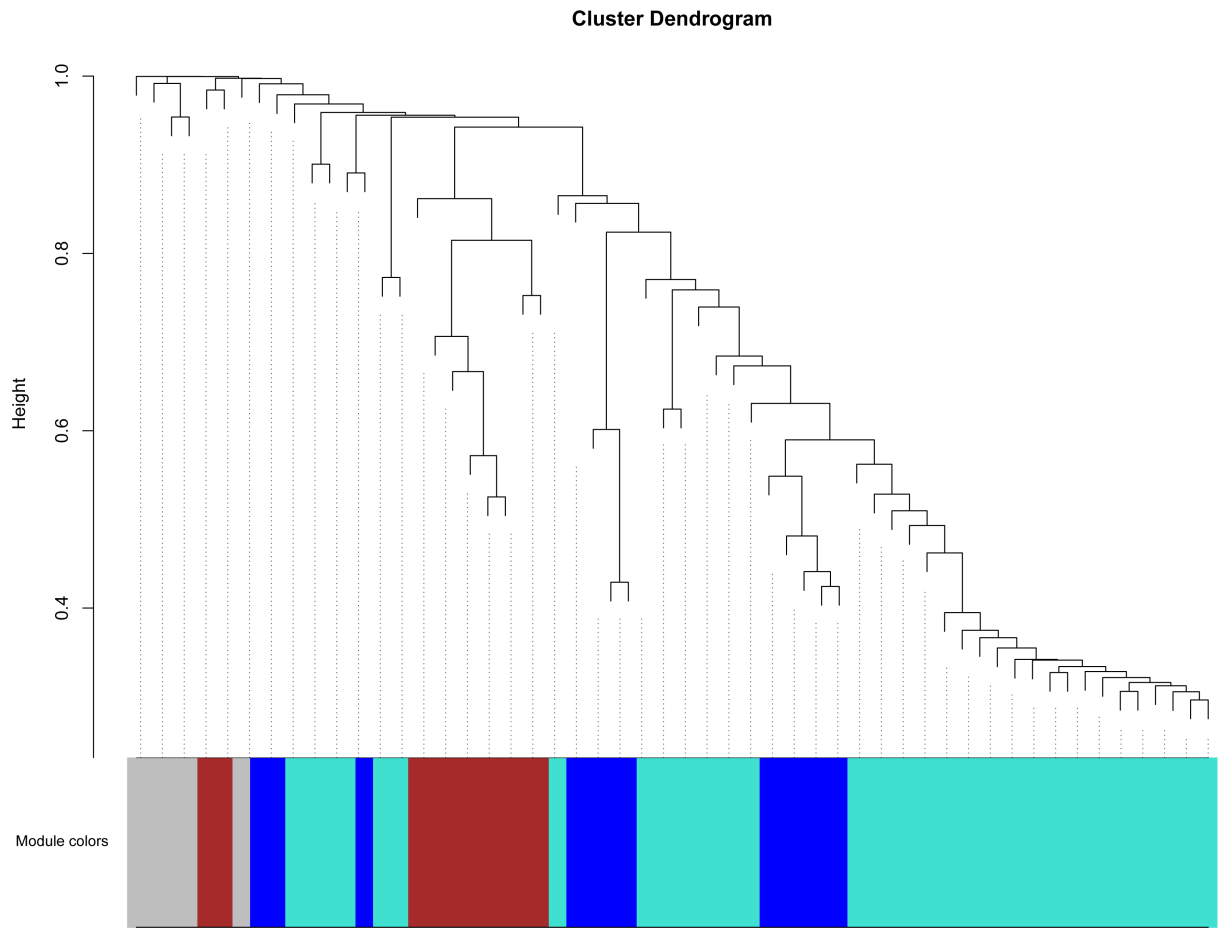

Supplement: Additional file 1: — Cluster dendrogram visualizing the modules from WGCNA. a Genes, b Metabolites were clustered using the average linkage hierarchical clustering and modules identified in the resulting dendrogram by the dynamic hybrid tree cut method. (DOCX 530 kb) [file 12859_2016_1045_MOESM1_ESM.docx]

**a**
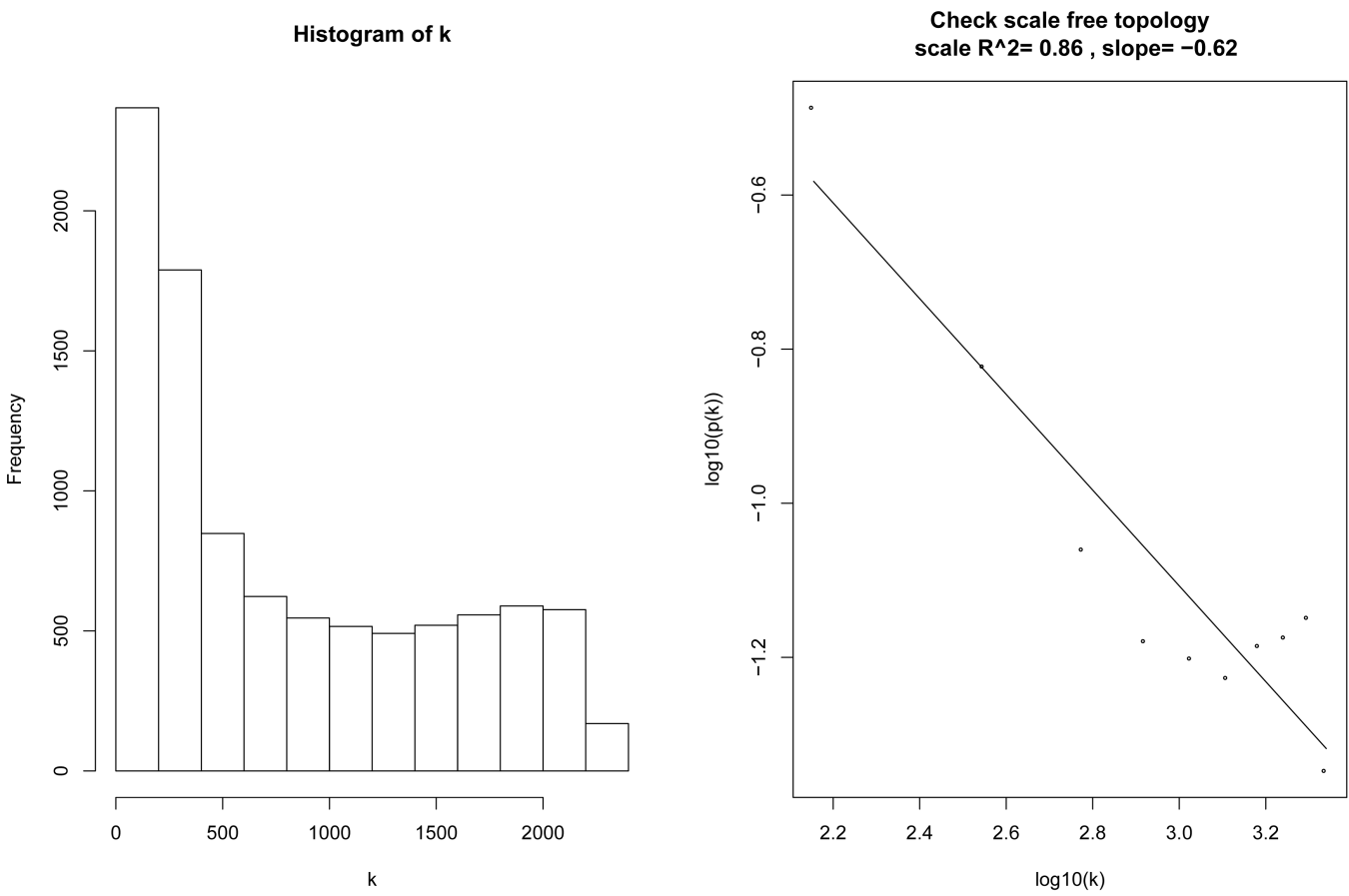


**b**


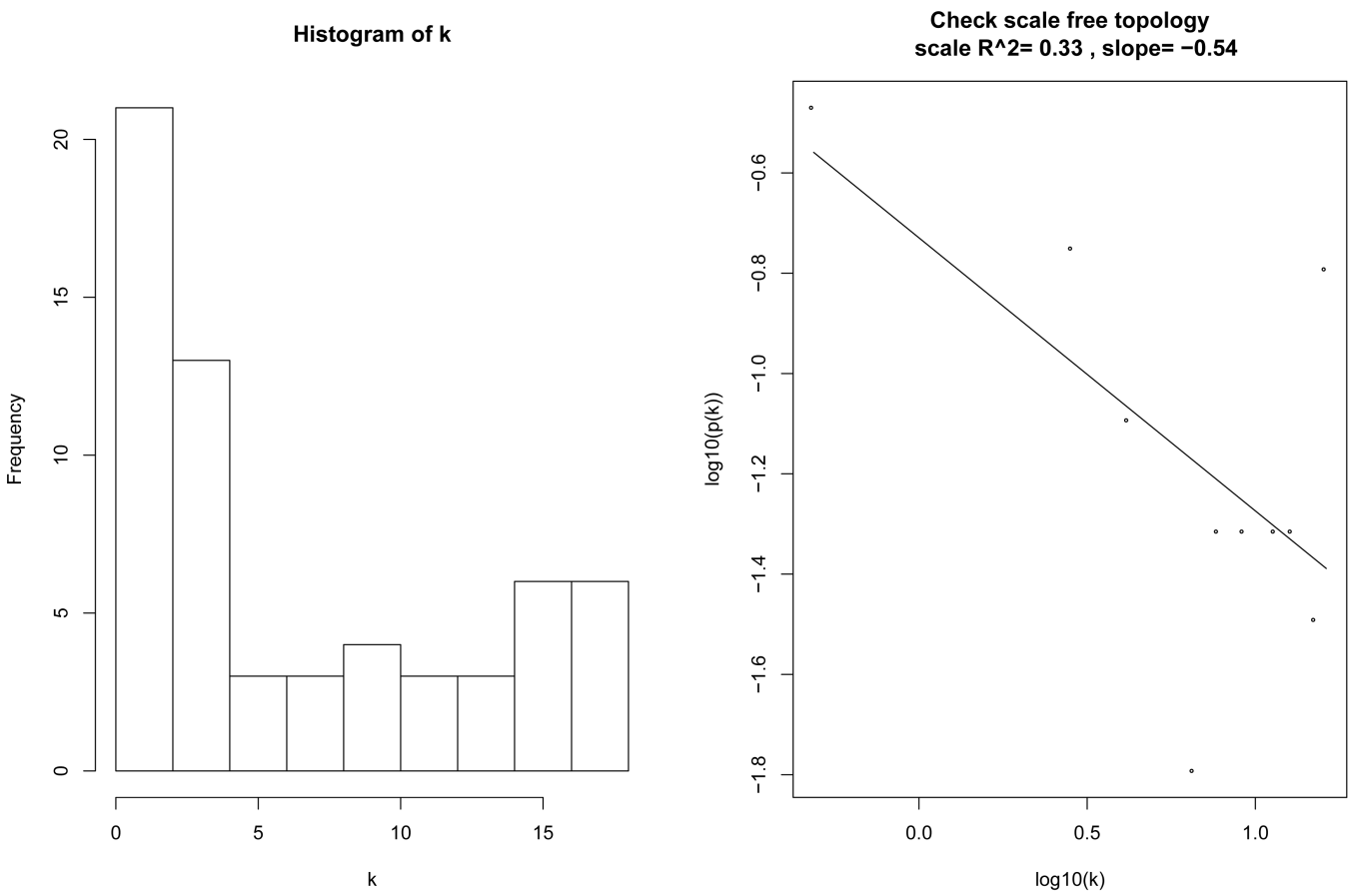

Supplement: Additional file 2: — Scale-free topologies. a Genes, b Metabolites. The left panel shows a histogram of network connectivity. The right panel shows a log-log plot of the histogram. The approximate straight line relationship (high R2 value) shows approximate scale free topology. (DOCX 143 kb) [file 12859_2016_1045_MOESM2_ESM.docx]
