# Supplementary material for: Network and biosignature analysis for the integration of transcriptomic and metabolomic data to characterize leaf senescence process in sunflower
Source: BMC Bioinformatics. 2016 Jun 6;17(Suppl 5):174. doi: 10.1186/s12859-016-1045-2 (PMC4905614; doi:10.1186/s12859-016-1045-2)
Supplement: Additional file 4: — BioSignature Discoverer results on predicting senescence from integrated metabolomics and transcriptomics data in sunflower. (DOCX 38 kb) [file 12859_2016_1045_MOESM4_ESM.docx]

# BioSignature Discoverer results on predicting senescence from integrated metabolomics and transcriptomics data in sunflower

## Signatures

| First Element | Second Element | Arabidopsis ID | Description |
| --- | --- | --- | --- |
| Alanine | HeAn_C_267 |  |  |
| HeAn_C_1048 | HeAn_C_267 | AT1G64640 | plastocyanin-like domain-containing protein |
| HeAn_C_11045 | HeAn_C_267 | AT5G58070 | TIL (TEMPERATURE-INDUCED LIPOCALIN |
| HeAn_C_11058 | HeAn_C_267 | AT2G02990 | RNS1 (RIBONUCLEASE 1) |
| HeAn_C_11653 | HeAn_C_267 | AT2G42690 | lipase, putative |
| HeAn_C_243 | HeAn_C_267 | AT5G45750 | Arabidopsis Rab GTPase homolog A1c |
| HeAn_C_3359 | HeAn_C_267 | AT4G22740 | glycine-rich protein |
| HeAn_C_8838 | HeAn_C_267 | AT1G55740 | Arabidopsis thaliana seed imbibition 1; hydrolase |
| HeAn_S_19086 | HeAn_C_267 | AT5G57320 | villin, putative |
| HeAn_S_20155 | HeAn_C_267 | AT4G13400 | unknown protein |
| HeAn_S_30642 | HeAn_C_267 | AT5G67070 | ralf-like 34; signal transducer |
| HeAn_S_35632 | HeAn_C_267 | AT2G47870 | glutaredoxin family protein |

## Predictive performances

| Metric | In Sample | Out Sample (days) | 95% Confidence Interval |
| --- | --- | --- | --- |
| R-squared | 0.9998 | 0.941 | [ 0.838, 0.970 ] |
| Mean Absolute Error | 0.104 | 1.974 | [ 1.249, 2.991 ] |
| Mean Squared Error | 0.0108 | 4.520 | [ 1.911, 8.552 ] |

## Residual Plot


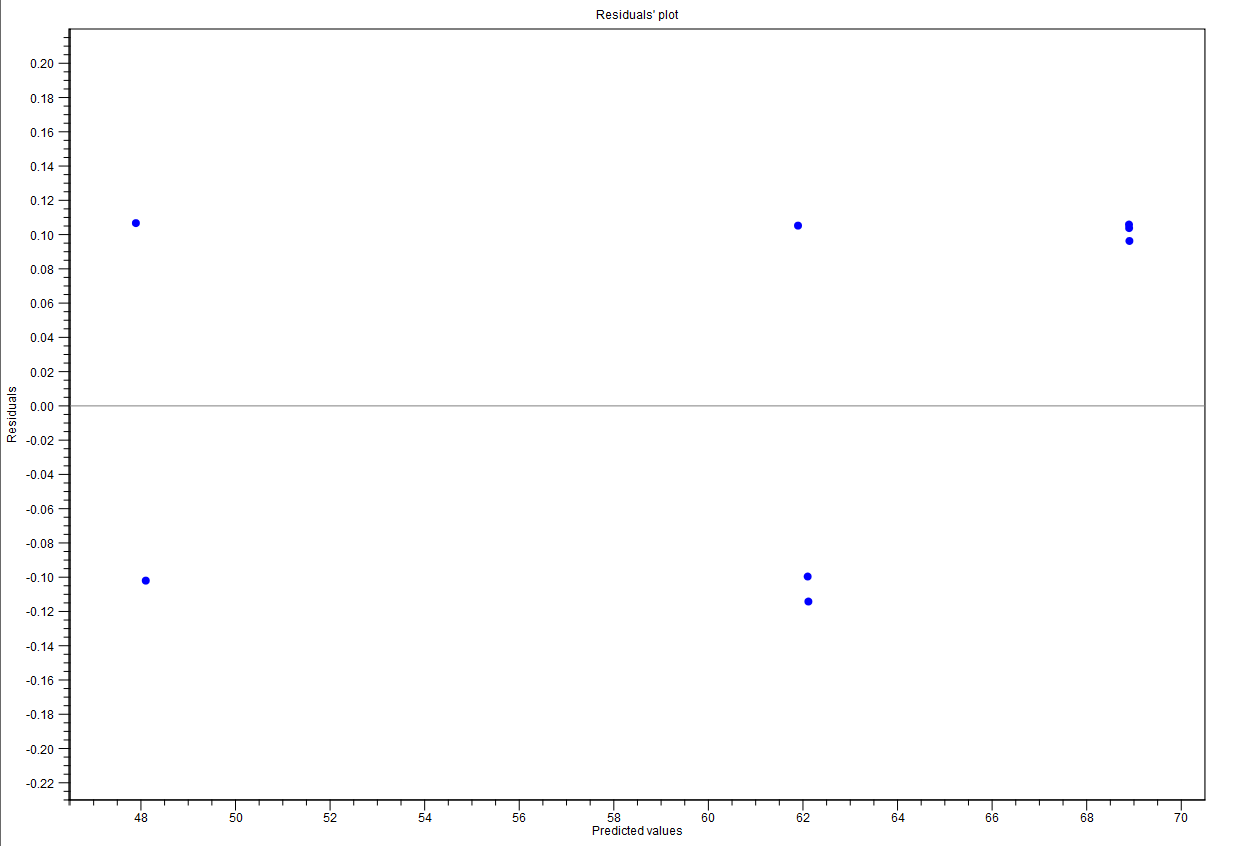


Figure 1: predicted values vs. residuals
